# Supplementary material for: The carbonization of aromatic molecules with three-dimensional structures affords carbon materials with controlled pore sizes at the Ångstrom-level
Source: Commun Chem. 2021 May 21;4:75. doi: 10.1038/s42004-021-00515-0 (PMC9814289; doi:10.1038/s42004-021-00515-0)
Supplement: Supplementary file 1 — Supplementary Information [file 42004_2021_515_MOESM1_ESM.pdf]

# Supporting Information

# The carbonization of aromatic molecules with three-dimensional structures affords carbon materials with controlled pore sizes at the Ångstrom-level

Tomoki Ogoshi,[1,2,*] Yuma Sakatsume,[3] Katsuto Onishi,[1] Rui Tang,[4] Kazuma Takahashi,[5] Hirotomo Nishihara,[4,5] Yuta Nishina,[6] Benoît D. L. Campéon,[6] Takahiro Kakuta[2,3] and Tada-aki Yamagishi[3]

[1]Department of Synthetic Chemistry and Biological Chemistry, Graduate School of Engineering, Kyoto University, Katsura, Nishikyo-ku, Kyoto 615-8510, Japan
[2]WPI Nano Life Science Institute (WPI-NanoLSI), Kanazawa University, Kakuma-machi, Kanazawa, 920-1192, Japan
[3]Graduate School of Natural Science and Technology, Kanazawa University, Kakuma-machi, Kanazawa, 920-1192, Japan
[4]Advanced Institute for Materials Research (WPI-AIMR), Tohoku University, 2-1-1 Katahira, Aoba-ku, Sendai, Miyagi, Japan
[5]Institute of Multidisciplinary Research for Advanced Materials, Tohoku University, 2-1-1 Katahira, Aoba-ku, Sendai, Miyagi, Japan
[6]Research Core for Interdisciplinary Sciences, Okayama University, 3-1-1 Tsushima-naka, Kita-ku, Okayama, Japan

*Tomoki Ogoshi: E-mail: ogoshi@sbchem.kyoto-u.ac.jp

**Supplementary Methods**

## Synthesis

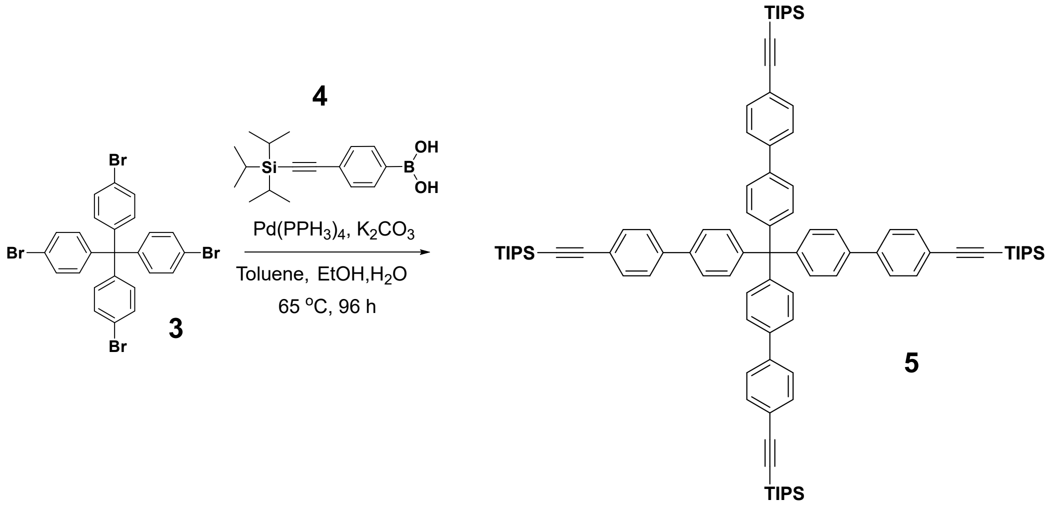


**Supplementary Fig. 1** Synthesis of **5**.

**5.** To a solution of tetrakis(4-bromophenyl)methane (**3**, 50 mg, 0.0800 mmol) in a mixture of toluene (5 mL), ethanol (1.5 mL) and water (1 mL), (4-((triisopropylsilyl)ethynyl)phenyl) boronic acid (**4**, 200 mg, 0.650 mmol), $Na_2CO_3$ (135 mg, 1.12 mmol) and $Pd(PPh_3)_4$ (14 mg, 0.0100 mmol) were added (**Supplementary Fig. 1**). The reaction mixture was heated at 65 °C for 96 h. After removal of the solvent, the resulting solid was dissolved in chloroform. After filtration, solvents were evaporated to give a solid. Column chromatography (silica gel; *n*-hexane/dichloromethane = 4/1) afforded a white solid quantitatively (**4**, 223 mg, 0.170 mmol). $^1$H NMR ($CDCl_3$, 500 MHz, ppm): δ 7.55, 7.53 (d, 16H, phenyl), 7.54, 7.52 (d, 8H, phenyl), 7.41, 7.39 (d, 8H, phenyl), 1.54 (s, 12H, isopropyl), 1.13 (s, 72H, isopropyl). $^{13}$C NMR ($CDCl_3$, 125 MHz, ppm): δ 146.0, 140.3, 138.0, 132.5, 131.4, 126.7, 126.2, 122.4, 106.9, 91.3, 18.6, 11.4. HRAPCIMS: *m*/*z* Calcd for $C_{93}H_{117}Si_4$ $[M+H]^+$: 1345.8227, found 1345.8242.

**2.** To a solution of **4** (200 mg, 0.150 mmol) in THF (20 mL), TBAF (186 mg, 0.710 mmol) was added (**Supplementary Fig. 2**). The mixture was stirred at 0 °C for 1 h, then ethyl acetate (200 mL) was added to the mixture. The organic phase was washed with 1 M $HCl_{aq.}$ (200 mL × 2) $H_2O$ (200 mL × 2). The organic phases were dried over $Na_2SO_4$. After filtration, the solvent was removed in *vacuo*. The residue was then purified by column chromatography (silica gel; *n*-hexane/dichloromethane = 9/1 to 1/1) affording a white solid (68.1 mg, 0.0900 mmol, 63%). $^1$H NMR ($CDCl_3$, 500 MHz, ppm): δ 7.59, 7.57 (d, 8H, phenyl), 7.56, 7.55 (d, 8H, phenyl), 7.55, 7.53 (d, 8H, phenyl), 7.41, 7.39 (d, 8H, phenyl), 3.12 (s, 4H, alkyne). $^{13}$C NMR ($CDCl_3$, 125 MHz, ppm): δ 146.03, 140.83, 137.78, 132.58, 131.50, 126.84, 126.21, 121.01, 83.52, 77.78, 64.24. HRAPCIMS: *m*/*z* Calcd for $C_{57}H_{37}$ $[M+H]^+$: 721.2890, found 721.2875.

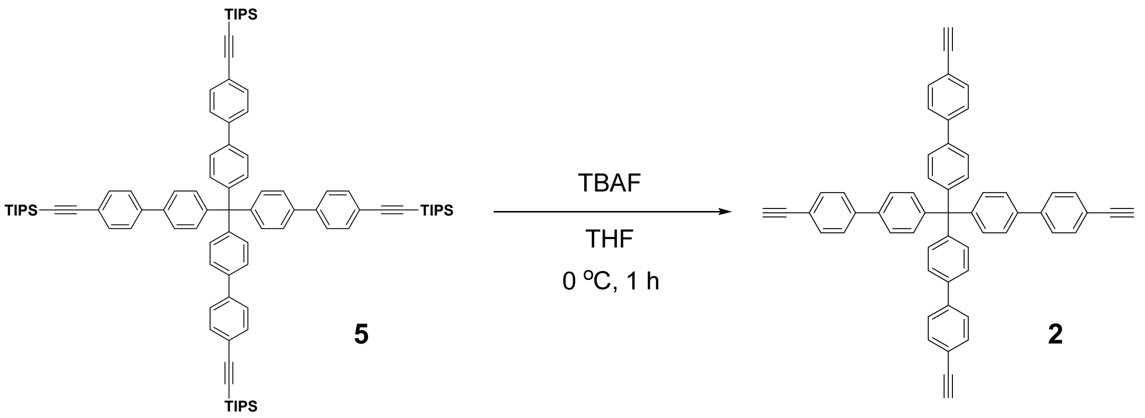


**Supplementary Fig. 2** Synthesis of **2**.

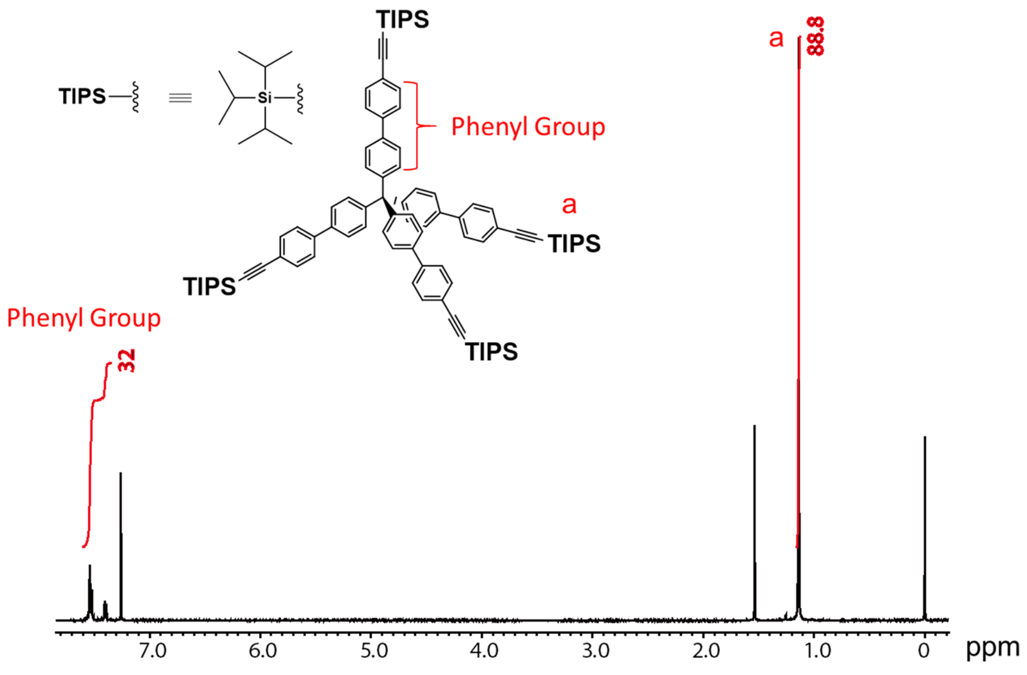


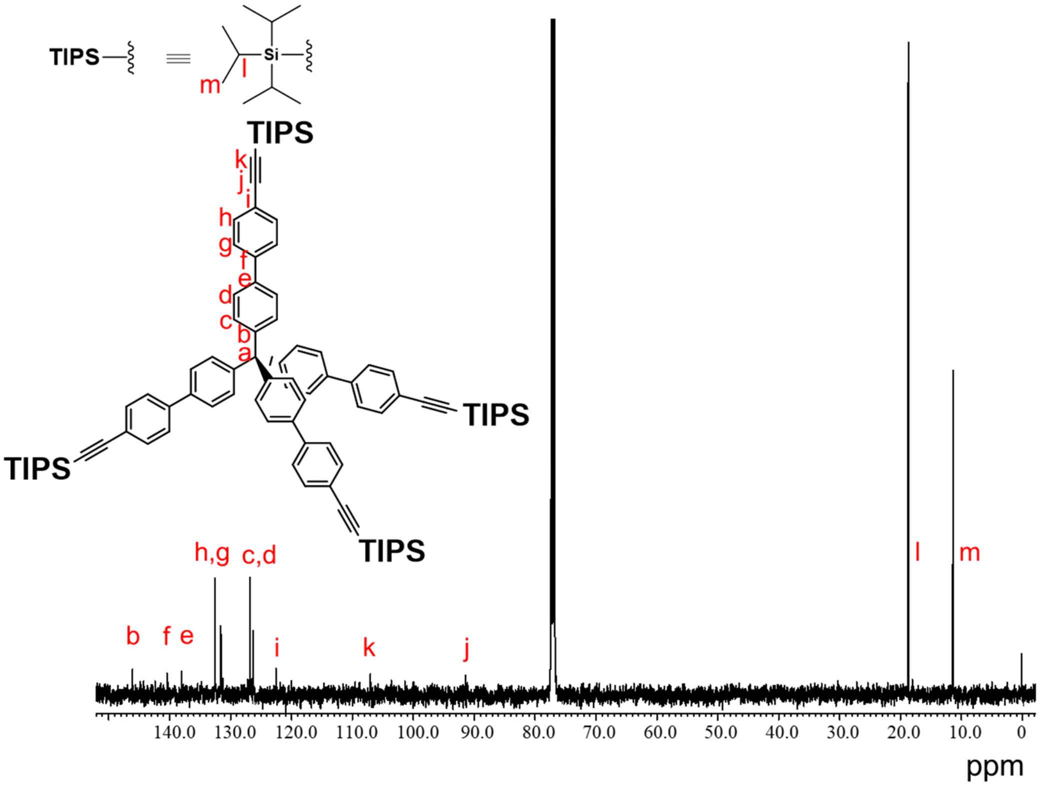


**Supplementary Fig. 3** (a) $^{1}$H and (b) $^{13}$C NMR spectra of **5** in $CDCl_3$ at 25 °C.

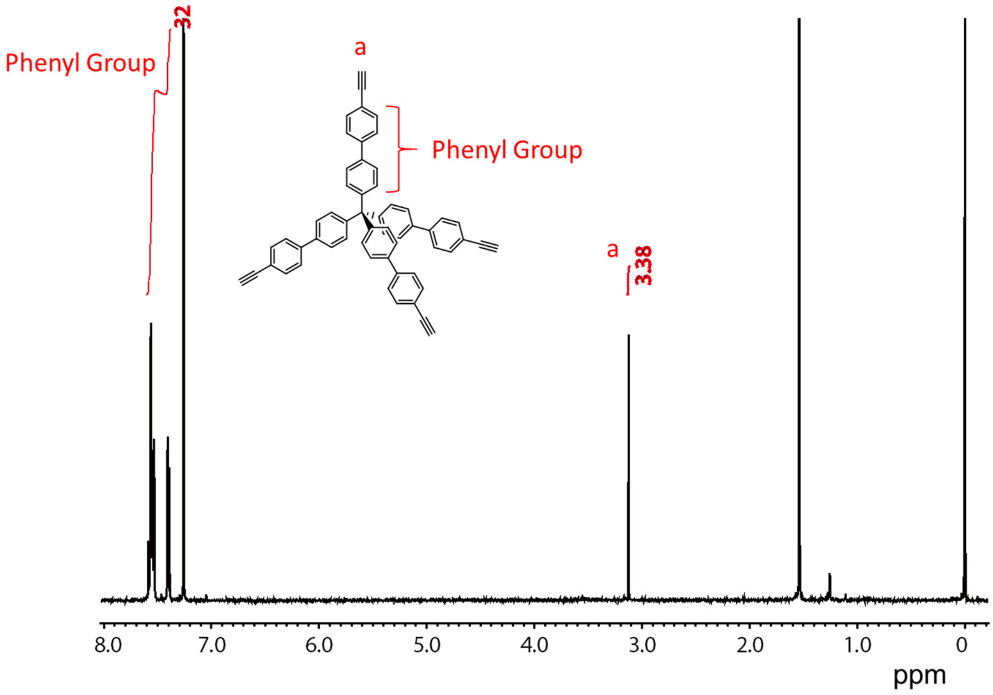


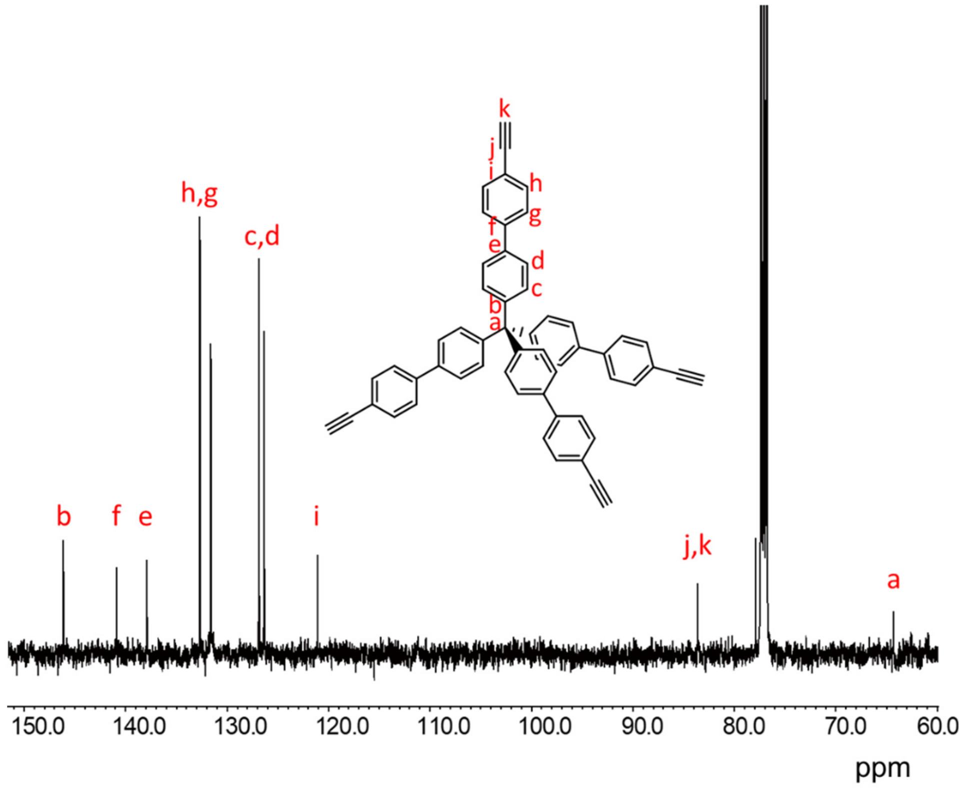


**Supplementary Fig. 4** (a) $^{1}$H and (b) $^{13}$C NMR spectra of **2** in $CDCl_3$ at 25 ºC.

**Carbonization**

The powders of **1**, **2** and **4** were heated under inert nitrogen atmosphere by temperature heating program as follows:

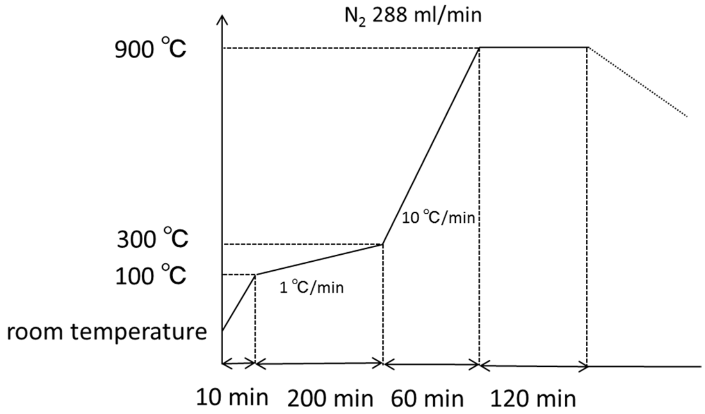


**Supplementary Fig. 5** Carbonization program of the carbon sources under nitrogen atmosphere.

Exothermic polymerization of the ethynyl groups of **1**, **2** and **4** during heating around 200-250 °C destroyed the carbons, resulting in low carbon yields. To avoid the decomposition of the product by exothermic polymerization, slow heating (1 °C/min) was carried out from 100 °C to 300 °C. In the final step, to carbonize the sample completely, the sample was heated at 900 °C for 120 min (**Supplementary Fig. 5**).

**MSC-30 Preparation**

MSC-30 was kindly provided by Kansai Coke and Chemicals Co., Ltd.

**ZTC Preparation**

ZTC was prepared by a traditional two-step method. Well-dried NaY zeolite ($SiO_2/Al_2O_3$ = 5.6, obtained from Tosoh Co., Ltd.) was impregnated with furfuryl alcohol, followed by washing with mesitylene. The zeolite powder accommodating furfuryl alcohol was heated at 150 °C for 8 h under $N_2$ flow to polymerize the monomer into polyfurfuryl alcohol. The resulting polymer/zeolite composite was heated up to 700 °C in $N_2$ flow with a heating rate of 5 °C/min. When the temperature becomes 700 °C, the $N_2$ gas was switched with a mixture of 7%-propylene/$N_2$, and chemical vapour deposition was carried out for 2 h. Then, the gas was switched back to $N_2$ and a post-treatment was performed at 900 °C for 3 h. After cooling down the sample, zeolite was dissolved away by hydrofluoric acid. Finally, the wet sample was dried at 150 °C

for 6 h under vacuum to obtain ZTC.

**LIB Preparation and Evaluation**

The CR2032 coin cells were assembled in an Ar-filled glove box to evaluate the electrochemical performance of **Cx** samples as anode materials for lithium-ion batteries. The slurry was prepared by mixing **Cx** (70%), carbon black (20%), and of poly(vinylidene fluoride) binder (10%) in an N-methylpyrrolidone (NMP) as a solvent. The anode was produced by coating the slurry onto copper foil as flat film with a thickness of 0.1 mm by doctor blade. Thin Lithium foil (0.6 mm thick) was employed as the counter electrode and a glass microfiber was used as the separator. The electrolyte was 1 M lithium hexafluorophosphate ($LiPF_6$), dissolved in 1/1 (V/V) ethylene carbonate (EC)/diethyl carbonate (DEC). The coin cells were tested in galvanostatic mode at various currents within a voltage range of 0.01 V to 3.0 V using a 580 Battery Test System (Toyo Corporation). The current rate was set as 1C = 372 mAh $g^{-1}$.

**SIB Preparation and Evaluation**

The CR2032 coin cells were assembled in an Ar-filled glove box to evaluate the electrochemical performance of **Cx** samples as anode materials for sodium-ion batteries. The slurry was prepared by mixing **Cx** (70%), carbon black (20%), and of poly(vinylidene fluoride) binder (10%) in an *N*-methylpyrrolidone (NMP) as a solvent. The anode was produced by coating the slurry onto copper foil as flat film with a thickness of 0.1 mm by doctor blade. Thin sodium foil (0.6 mm thick) was employed as the counter electrode and a glass microfiber was used as the separator. The electrolyte was 1 M sodium hexafluorophosphate ($NaPF_6$), dissolved in 1/1 (V/V) ethylene carbonate (EC)/diethyl carbonate (DEC). The coin cells were tested in galvanostatic mode at various currents within a voltage range of 0.01 V to 3.0 V using a 580 Battery Test System (Toyo Corporation). The current rate was set as 1C = 372 mAh $g^{-1}$.

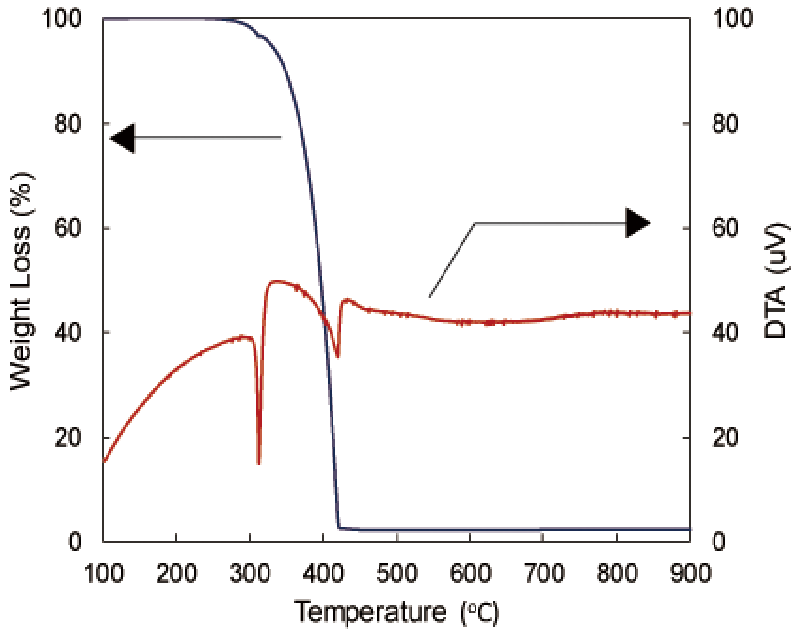


**Supplementary Fig. 6** Thermo-gravimetric analysis (TGA, blue line) and differential thermal analysis (DTA, red line) of tetrakis(4-bromophenyl)methane (without ethynyl groups) **3** under nitrogen atmosphere (heating rate: 10 °C / min).

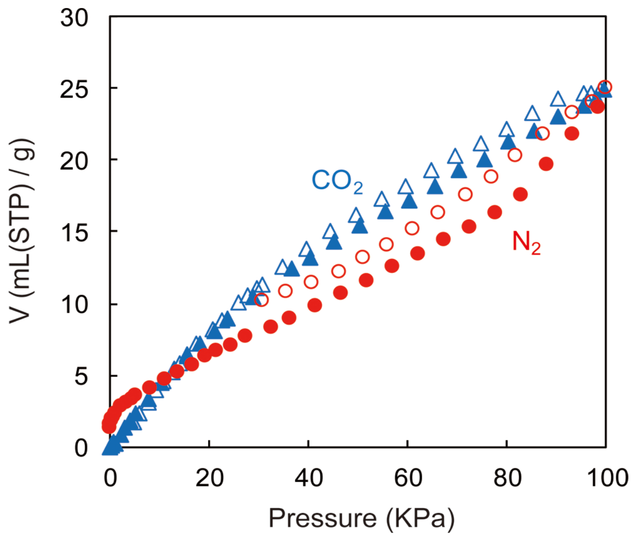


**Supplementary Fig. 7** $CO_2$ (25 °C, blue triangles) and $N_2$ (- 196 °C, red circles) sorption isotherms of the powders of **1** after the thermal polymerization at 300 °C. Solid symbols = adsorption; open symbols = desorption.

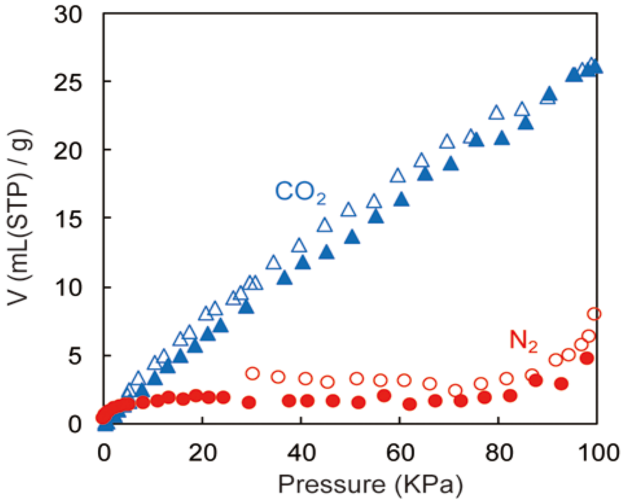


**Supplementary Fig. 8** $CO_2$ (25 °C, blue triangles) and $N_2$ (- 196 °C, red circles) sorption isotherms of the powders of **2** after the thermal polymerization at 300 °C. Solid symbols = adsorption; open symbols = desorption.

**Supplementary Table 1**. The amounts of adsorption gases from the samples during the TPD analysis and the CHO ratios calculated from the amounts of the adsorption gases.

| **Sample** | **$H_2$ / µmol g$^{-1}$** | **$H_2O$ / µmol g$^{-1}$** | **CO / µmol g$^{-1}$** | **H / wt%** | **O / wt%** | **C / wt%** |
|---|---|---|---|---|---|---|
| **C1** | **1347** | **325** | **449** | **0.34** | **1.68** | **97.98** |
| **C2** | **1335** | **194** | **417** | **0.31** | **1.36** | **98.33** |

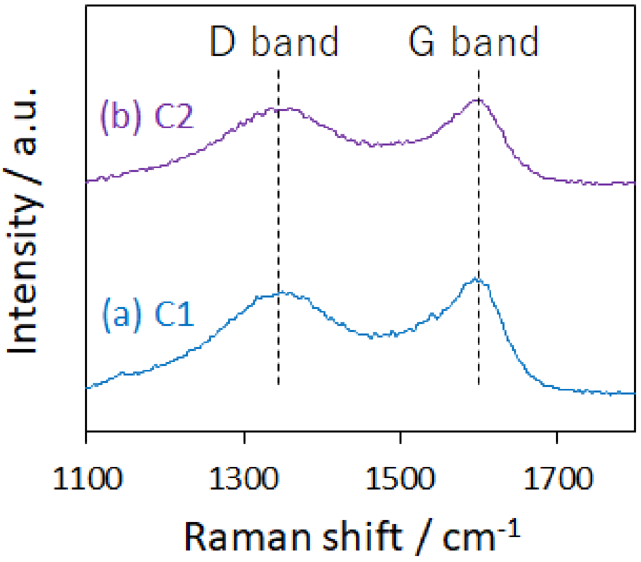


**Supplementary Fig. 9** The Raman pattern of carbons (a) **C1** and (b) **C2** showed peaks at 1355 and 1590 $cm^{-1}$, which correspond to the D and G bands, respectively.

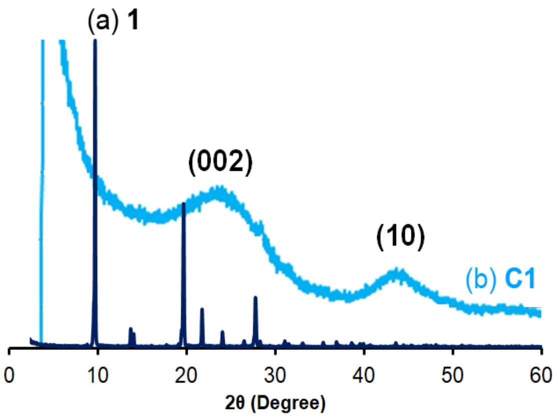


**Supplementary Fig. 10** Powder X-ray diffraction patterns of (a) **1** and (b) **C1**.

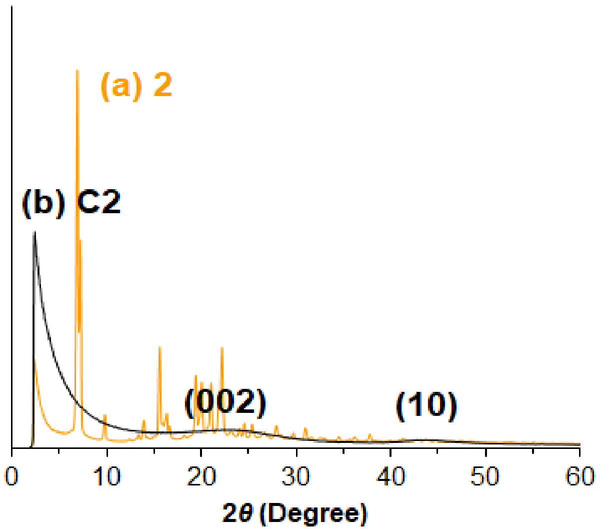


**Supplementary Fig. 11** Powder X-ray diffraction patterns of (a) **2** and (b) **C2**.

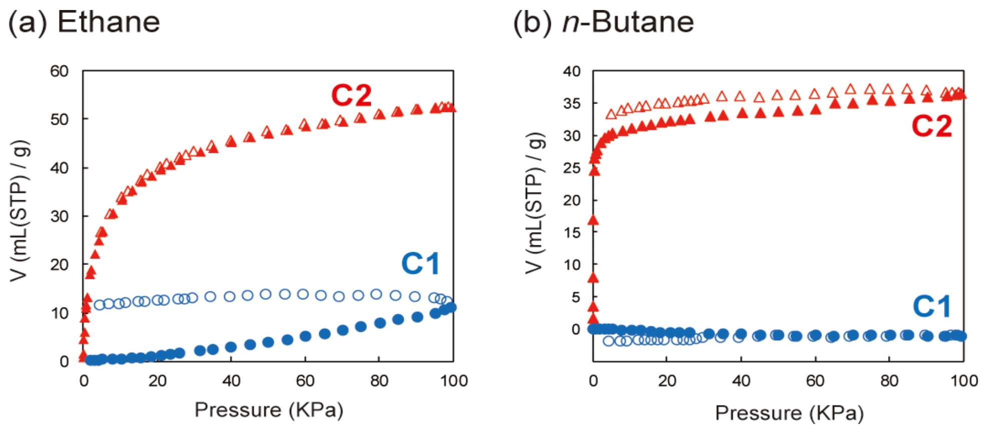


**Supplementary Fig. 12** (a) Ethane (25 ºC) and (b) *n*-butane (25 ºC) sorption isotherms of the powders of porous carbons **C1** (blue circles) and **C2** (red triangles). Solid symbols = adsorption; open symbols = desorption.

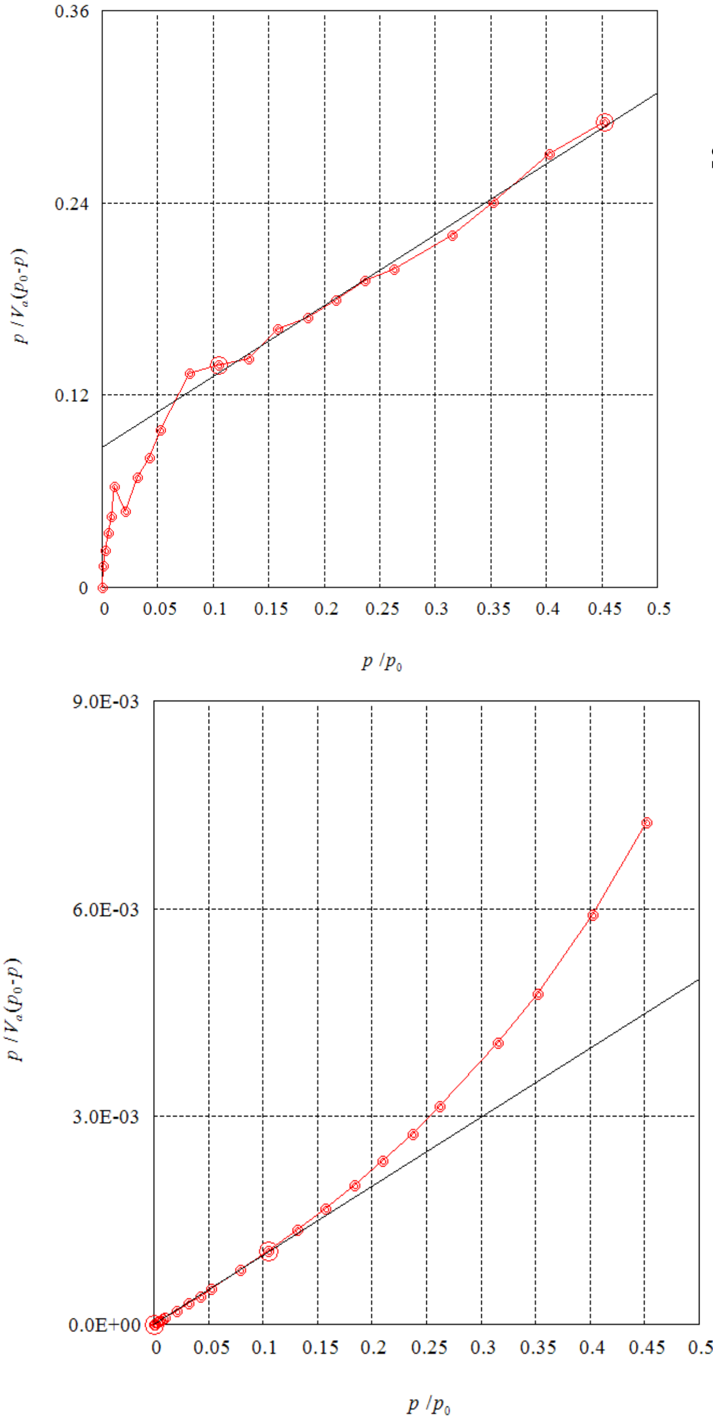


Surface Area: 8.21 $m^2/g$

Surface Area: $4.37 \times 10^2$ $m^2/g$

**Supplementary Fig. 13** BET plots of **C1** and **C2** using $N_2$ sorption isotherms.

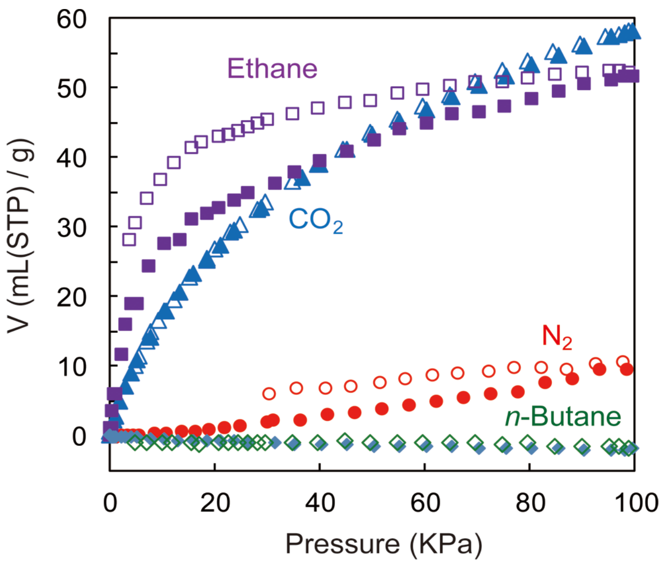


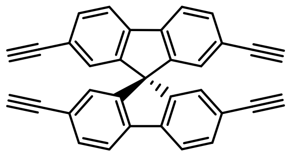

**4**

**Supplementary Fig. 14** Ethane (25 ºC, purple squares), $CO_2$ (25 ºC, blue triangles), $N_2$ (- 196 ºC, red circles) and *n*-butane (25 ºC, green diamonds) sorption isotherms of the powders of porous carbons **C4**. Solid symbols = adsorption; open symbols = desorption.

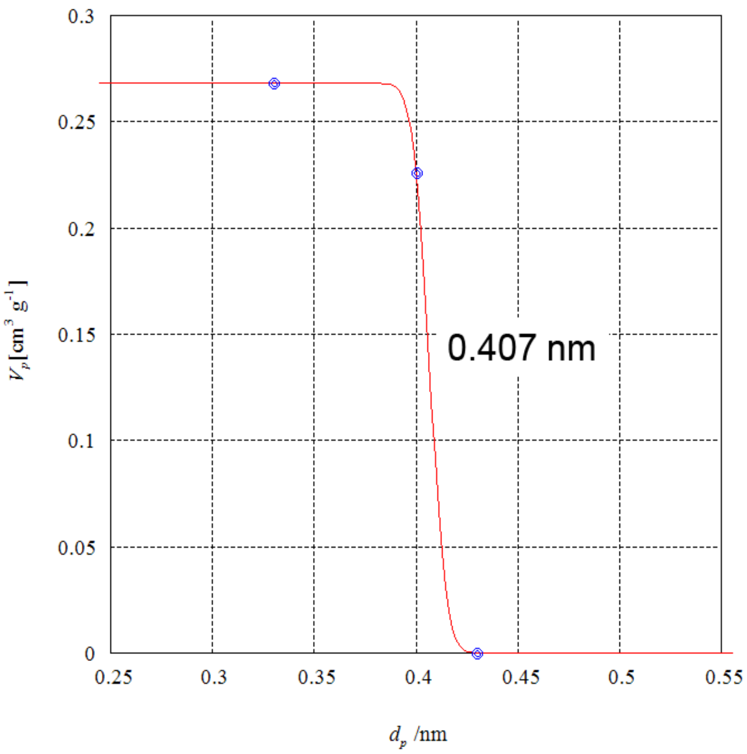


**Supplementary Fig. 15** Micropore volume (MV) of **C4** estimated by the DA method, plotted against kinetic diameter of molecular probes.

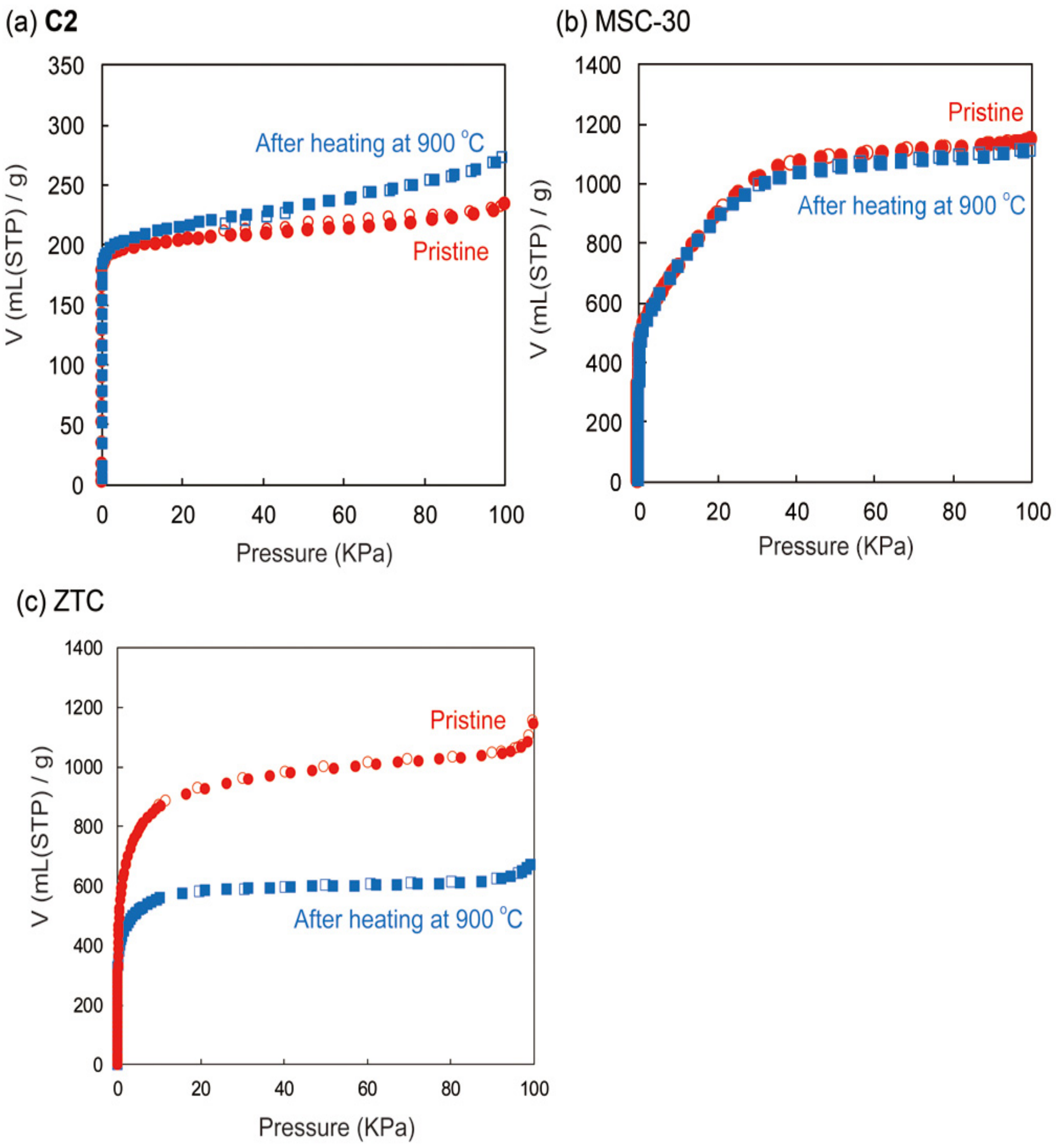


**Supplementary Fig. 16** $N_2$ (- 196 ºC) sorption isotherms of the powders of (a) **C2**, (b) MSC-30 and (c) ZTC before (red circles) and after heating at 900 ºC (blue squares). Solid symbols = adsorption; open symbols = desorption.

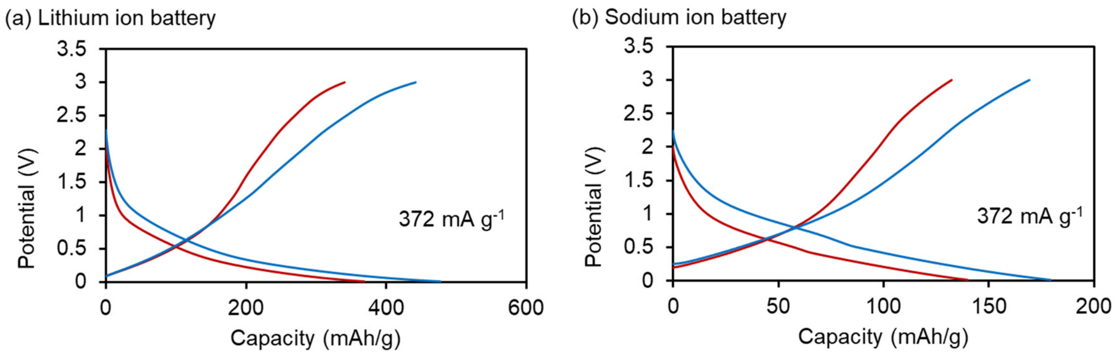


**Supplementary Fig. 17** Charge discharge performance of **C1** (blue) and **C2** (red) for anode active materials (a) for lithium and (b) sodium ion batteries.

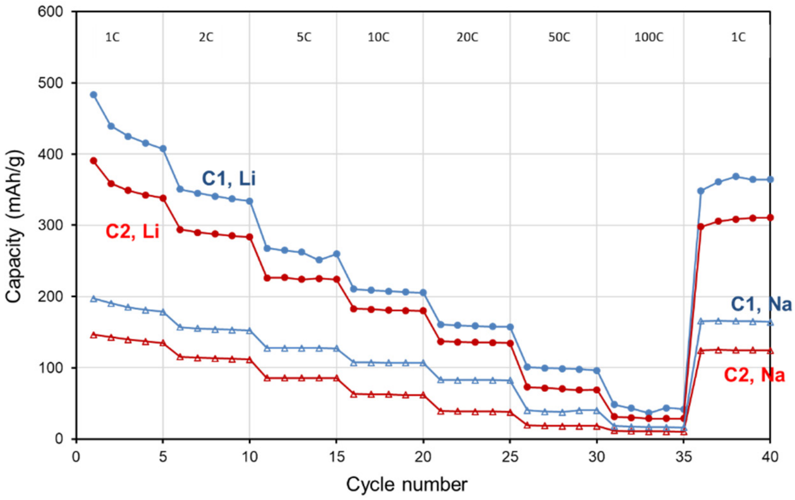


**Supplementary Fig. 18** Application of **C1** (blue) and **C2** (red) for anode active materials for lithium (circles) and sodium (triangles) ion batteries.
